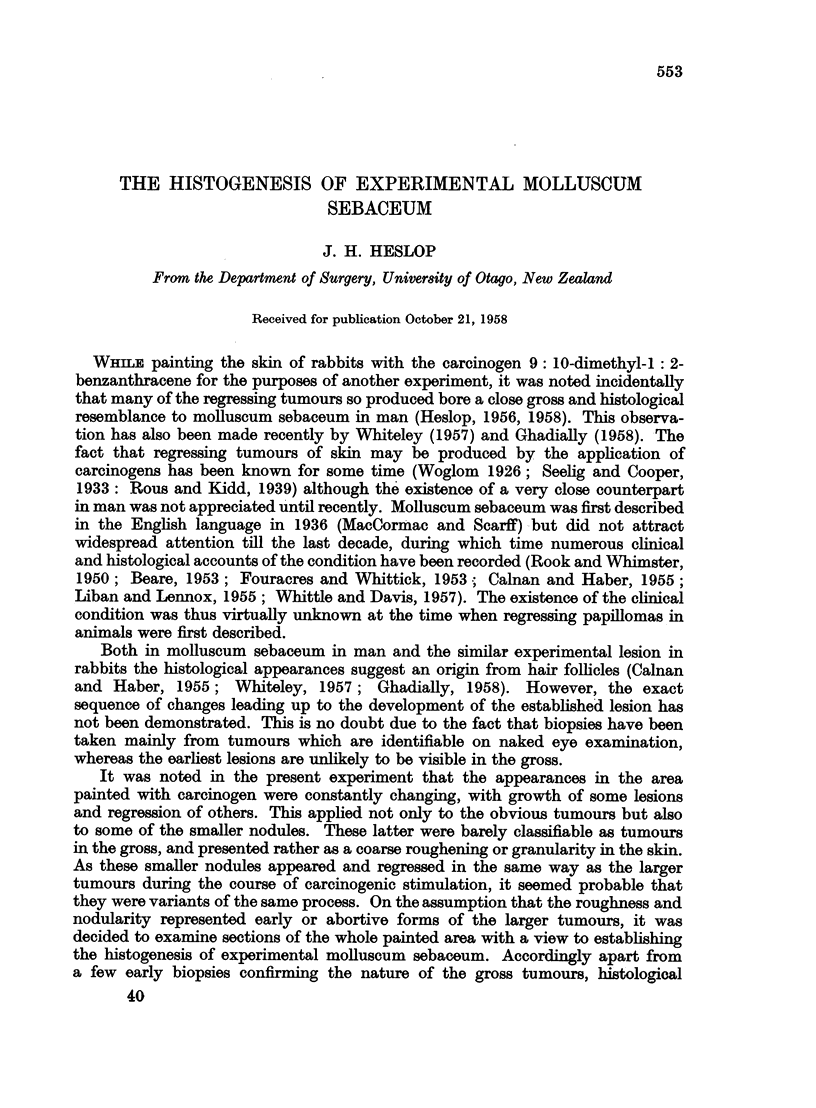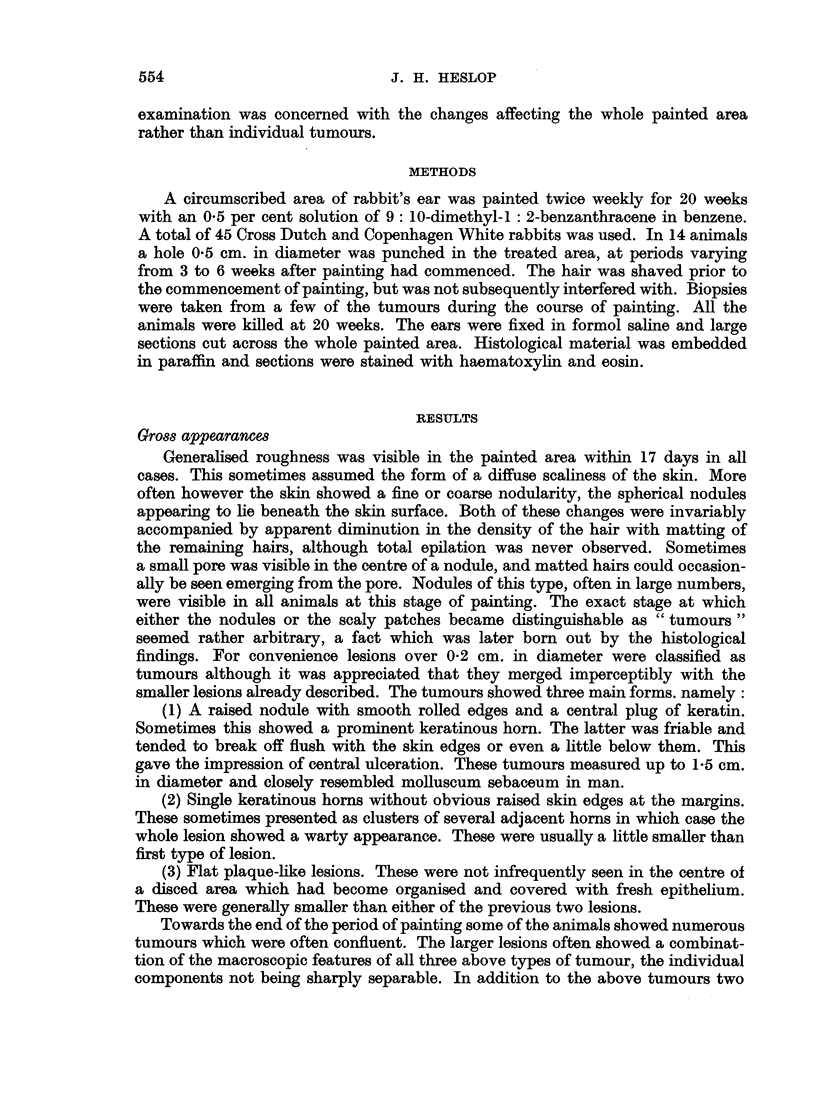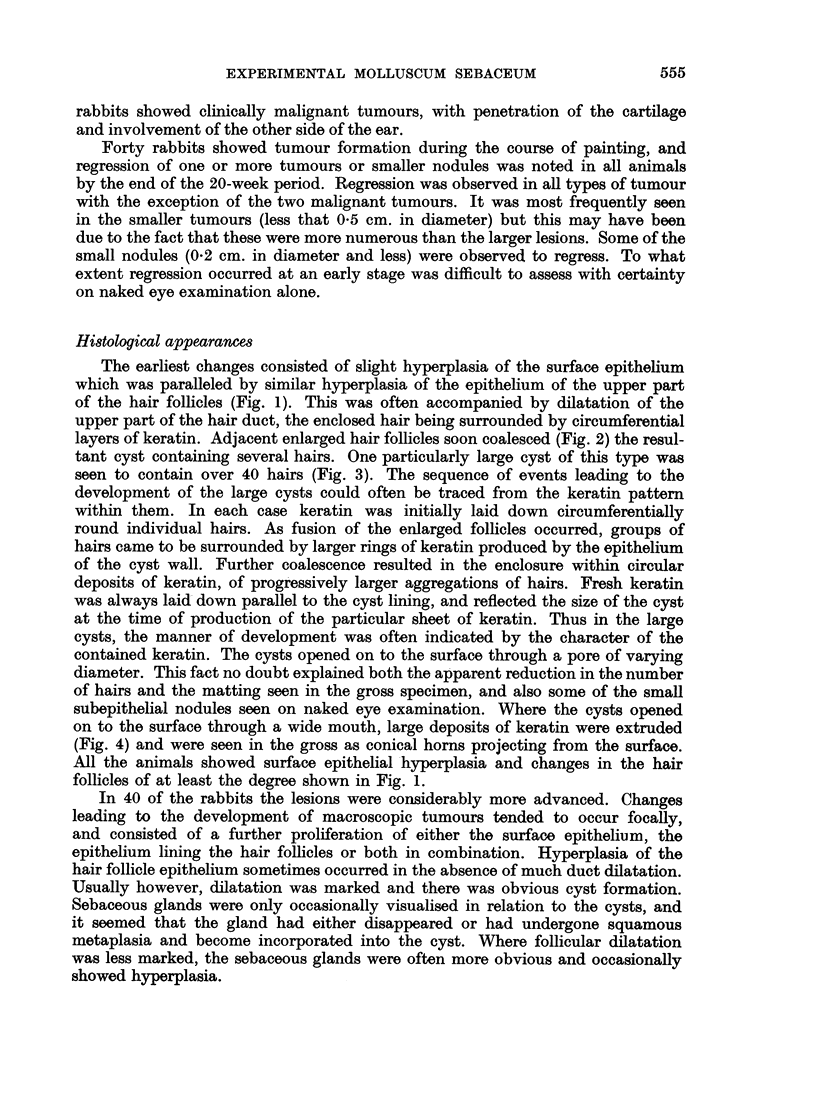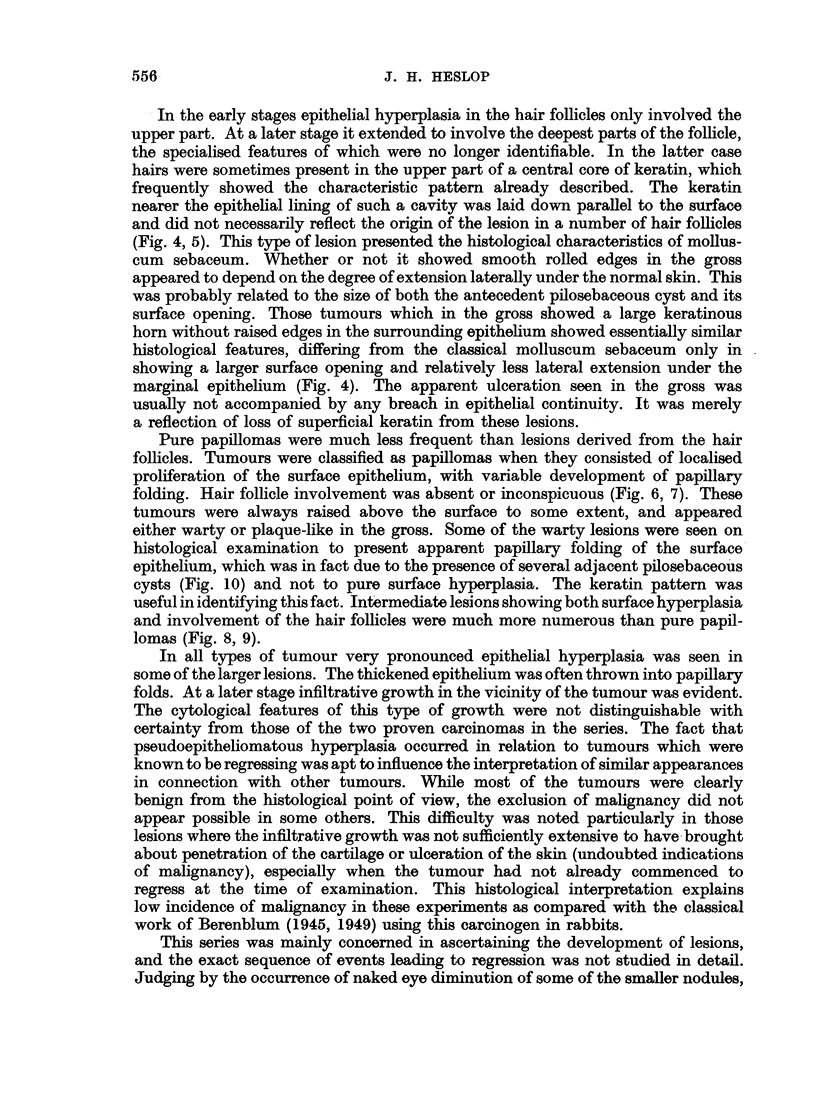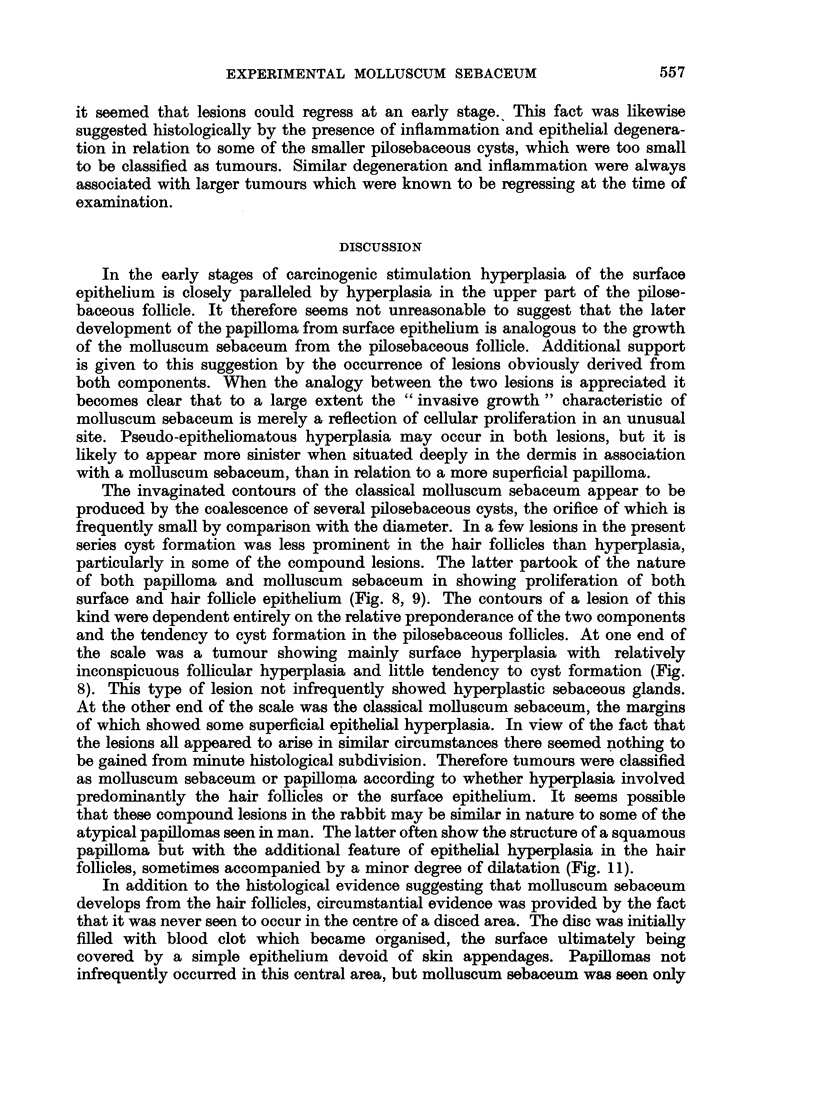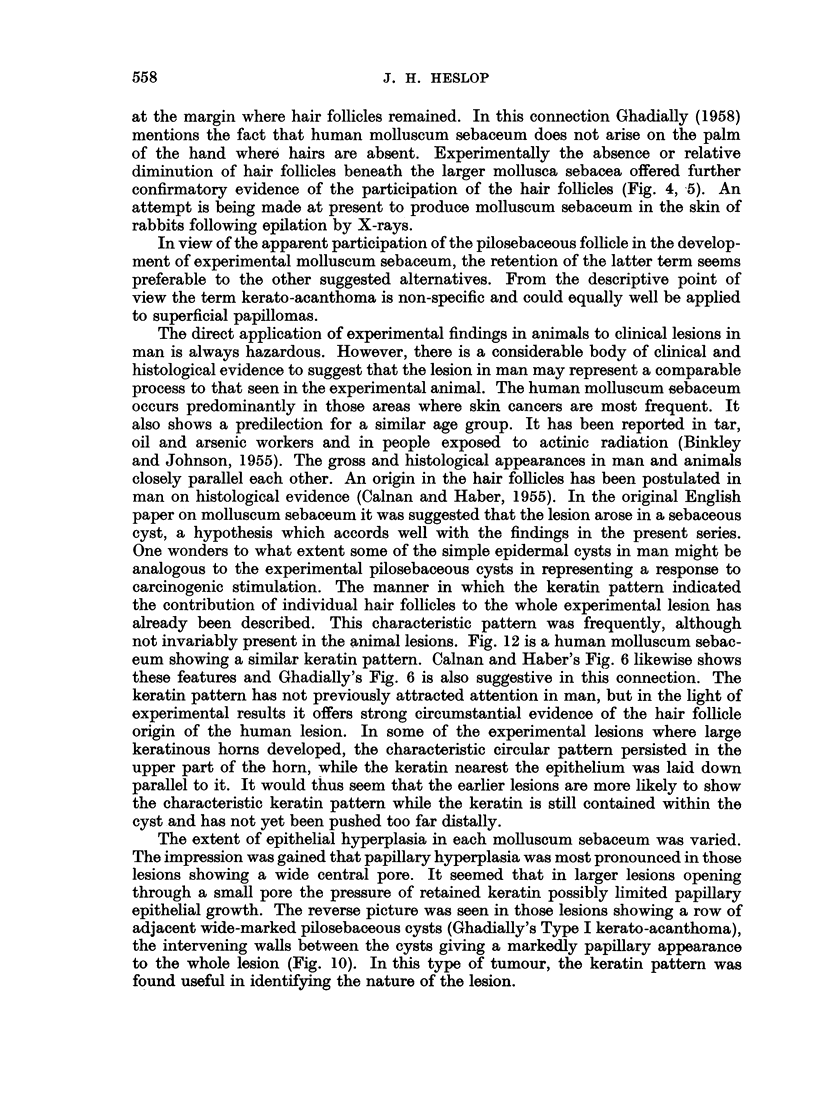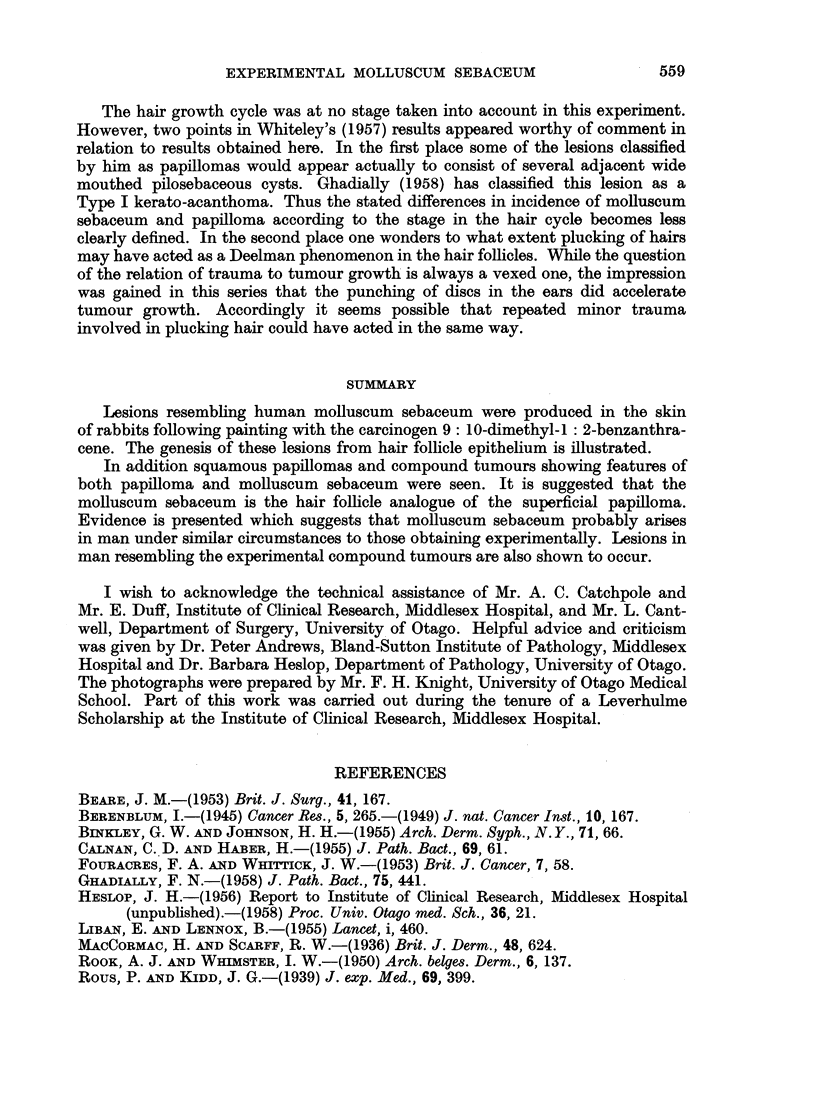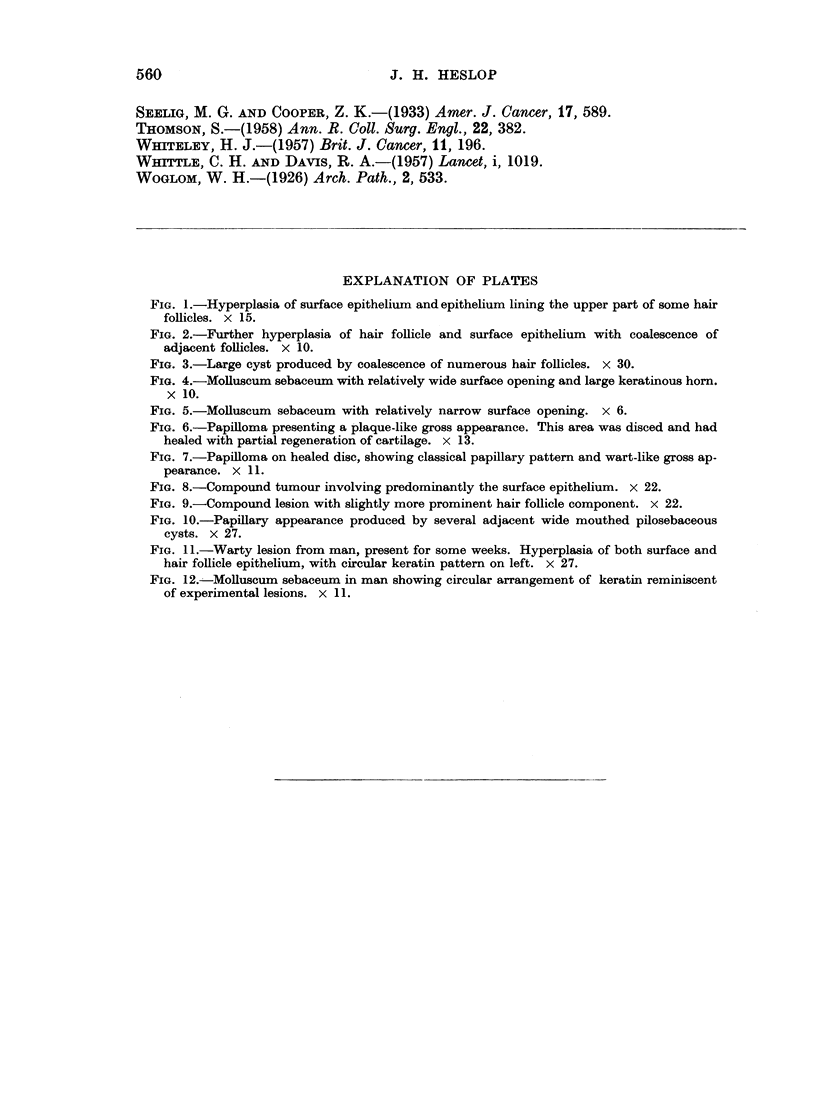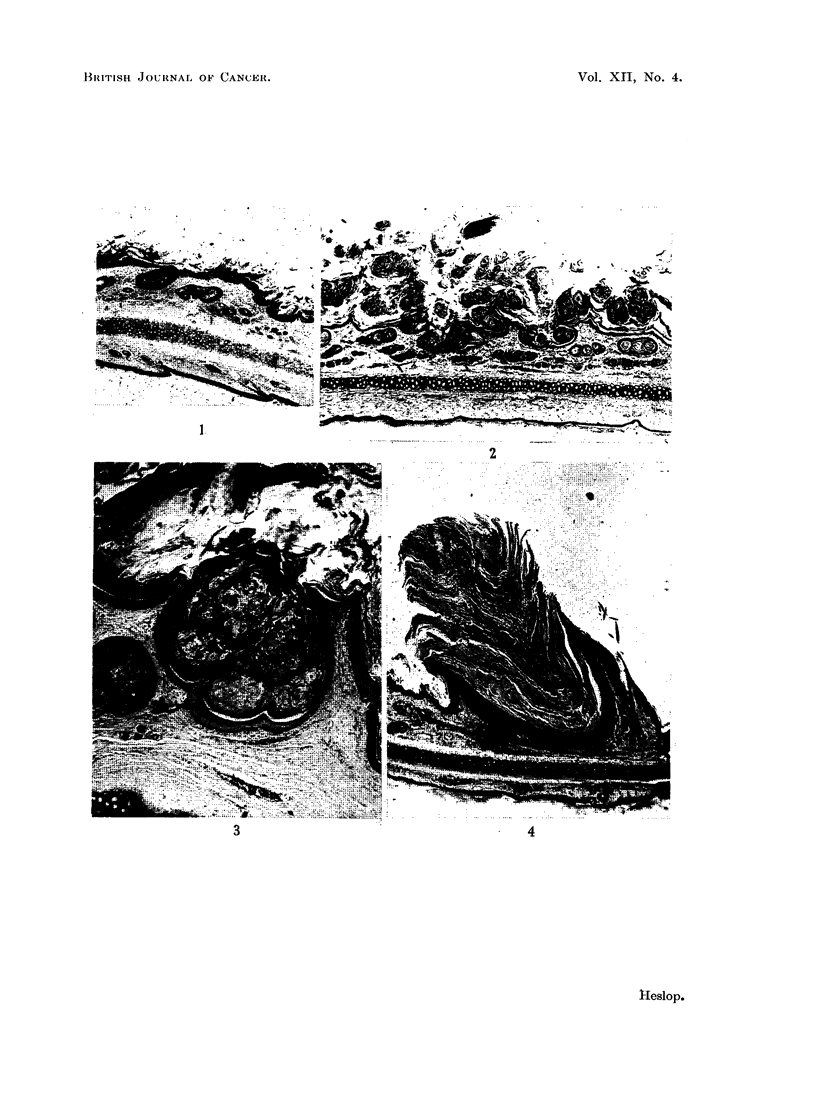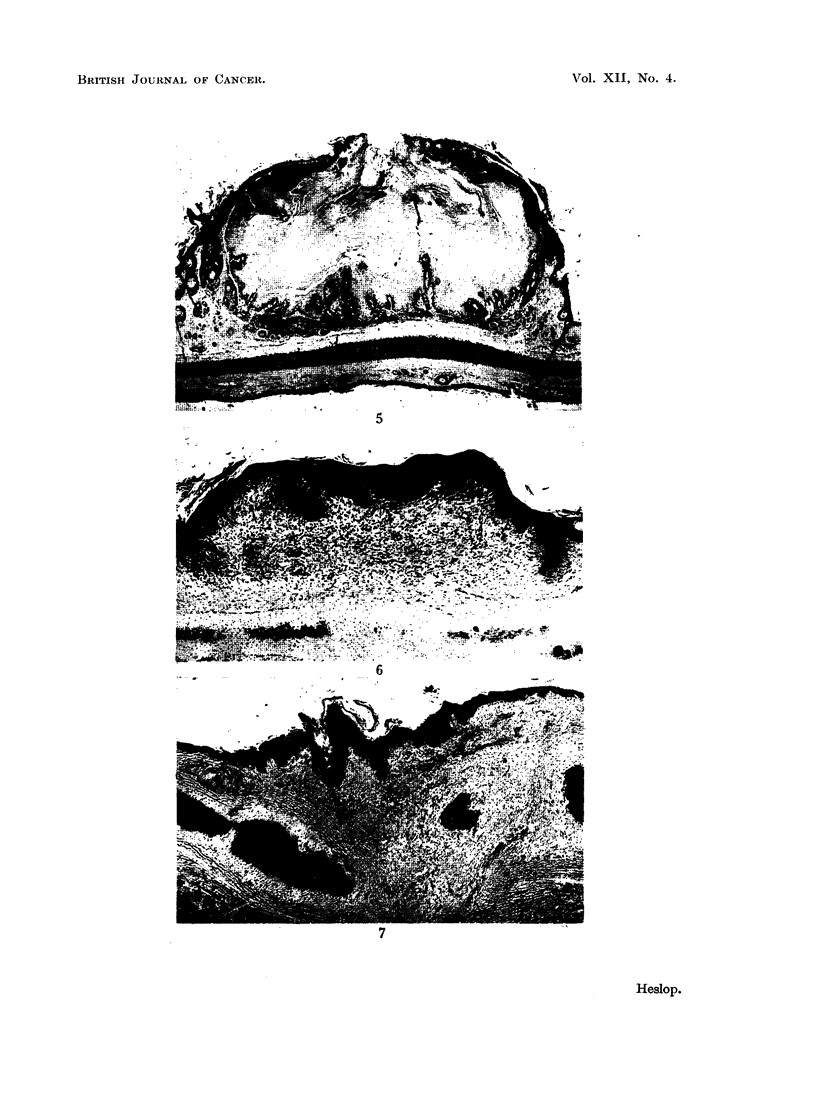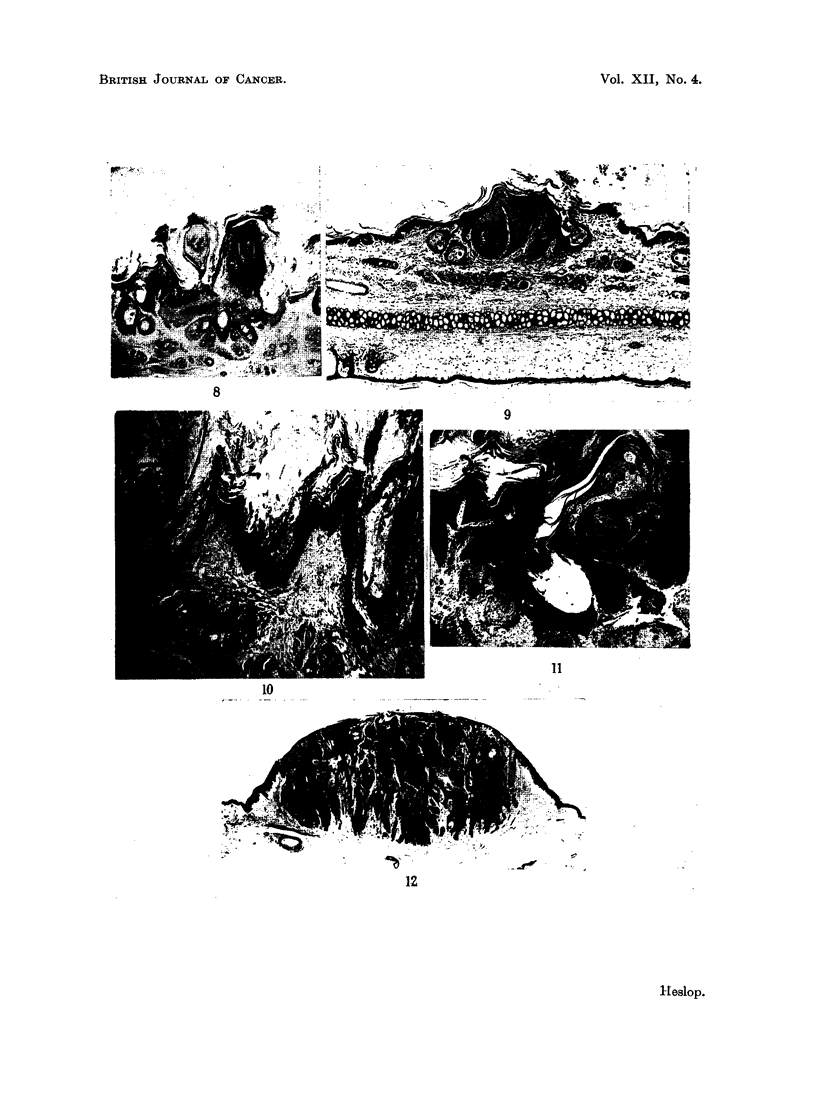# The Histogenesis of Experimental Molluscum Sebaceum

**DOI:** 10.1038/bjc.1958.64

**Published:** 1958-12

**Authors:** J. H. Heslop

## Abstract

**Images:**


### THE HISTOGENESIS OF EXPERIMENTAL MOLLUSCUM SEBACEUM

J. H. HESLOP

*From the Department of Surgery, University of Otago, New Zealand*

Received for publication October 21, 1958

WHILE painting the skin of rabbits with the carcinogen 9 : 10-dimethyl-1 : 2-benzanthracene for the purposes of another experiment, it was noted incidentally that many of the regressing tumours so produced bore a close gross and histological resemblance to molluscum sebaceum in man (Heslop, 1956, 1958). This observation has also been made recently by Whiteley (1957) and Ghadially (1958). The fact that regressing tumours of skin may be produced by the application of carcinogens has been known for some time (Woglom 1926 ; Seelig and Cooper, 1933 : Rous and Kidd, 1939) although the existence of a very close counterpart in man was not appreciated until recently. Molluscum sebaceum was first described in the English language in 1936 (MacCormac and Scarff) but did not attract widespread attention till the last decade, during which time numerous clinical and histological accounts of the condition have been recorded (Rook and Whimster, 1950 ; Beare, 1953 ; Fouracres and Whittick, 1953 ; Calnan and Haber, 1955 ; Liban and Lennox, 1955 ; Whittle and Davis, 1957). The existence of the clinical condition was thus virtually unknown at the time when regressing papillomas in animals were first described.

Both in molluscum sebaceum in man and the similar experimental lesion in rabbits the histological appearances suggest an origin from hair follicles (Calnan and Haber, 1955 ; Whiteley, 1957 ; Ghadially, 1958). However, the exact sequence of changes leading up to the development of the established lesion has not been demonstrated. This is no doubt due to the fact that biopsies have been taken mainly from tumours which are identifiable on naked eye examination, whereas the earliest lesions are unlikely to be visible in the gross.

It was noted in the present experiment that the appearances in the area painted with carcinogen were constantly changing, with growth of some lesions and regression of others. This applied not only to the obvious tumours but also to some of the smaller nodules. These latter were barely classifiable as tumours in the gross, and presented rather as a coarse roughening or granularity in the skin. As these smaller nodules appeared and regressed in the same way as the larger tumours during the course of carcinogenic stimulation, it seemed probable that they were variants of the same process. On the assumption that the roughness and nodularity represented early or abortive forms of the larger tumours, it was decided to examine sections of the whole painted area with a view to establishing the histogenesis of experimental molluscum sebaceum. Accordingly apart from a few early biopsies confirming the nature of the gross tumours, histological examination was concerned with the changes affecting the whole painted area rather than individual tumours.

### METHODS

A circumscribed area of rabbit's ear was painted twice weekly for 20 weeks with an 0·5 per cent solution of 9 : 10-dimethyl-1 : 2-benzanthracene in benzene. A total of 45 Cross Dutch and Copenhagen White rabbits was used. In 14 animals a hole 0·5 cm. in diameter was punched in the treated area, at periods varying from 3 to 6 weeks after painting had commenced. The hair was shaved prior to the commencement of painting, but was not subsequently interfered with. Biopsies were taken from a few of the tumours during the course of painting. All the animals were killed at 20 weeks. The ears were fixed in formol saline and large sections cut across the whole painted area. Histological material was embedded in paraffin and sections were stained with haematoxylin and eosin.

### RESULTS

### *Gross appearances*

Generalised roughness was visible in the painted area within 17 days in all cases. This sometimes assumed the form of a diffuse scaliness of the skin. More often however the skin showed a fine or coarse nodularity, the spherical nodules appearing to lie beneath the skin surface. Both of these changes were invariably accompanied by apparent diminution in the density of the hair with matting of the remaining hairs, although total epilation was never observed. Sometimes a small pore was visible in the centre of a nodule, and matted hairs could occasionally be seen emerging from the pore. Nodules of this type, often in large numbers, were visible in all animals at this stage of painting. The exact stage at which either the nodules or the scaly patches became distinguishable as " tumours " seemed rather arbitrary, a fact which was later born out by the histological findings. For convenience lesions over 0·2 cm. in diameter were classified as tumours although it was appreciated that they merged imperceptibly with the smaller lesions already described. The tumours showed three main forms. namely :

(1) A raised nodule with smooth rolled edges and a central plug of keratin. Sometimes this showed a prominent keratinous horn. The latter was friable and tended to break off flush with the skin edges or even a little below them. This gave the impression of central ulceration. These tumours measured up to 1·5 cm. in diameter and closely resembled molluscum sebaceum in man.

(2) Single keratinous horns without obvious raised skin edges at the margins. These sometimes presented as clusters of several adjacent horns in which case the whole lesion showed a warty appearance. These were usually a little smaller than first type of lesion.

(3) Flat plaque-like lesions. These were not infrequently seen in the centre of a disced area which had become organised and covered with fresh epithelium. These were generally smaller than either of the previous two lesions.

Towards the end of the period of painting some of the animals showed numerous tumours which were often confluent. The larger lesions often showed a combinattion of the macroscopic features of all three above types of tumour, the individual components not being sharply separable. In addition to the above tumours two rabbits showed clinically malignant tumours, with penetration of the cartilage and involvement of the other side of the ear.

Forty rabbits showed tumour formation during the course of painting, and regression of one or more tumours or smaller nodules was noted in all animals by the end of the 20-week period. Regression was observed in all types of tumour with the exception of the two malignant tumours. It was most frequently seen in the smaller tumours (less that 0·5 cm. in diameter) but this may have been due to the fact that these were more numerous than the larger lesions. Some of the small nodules (0·2 cm. in diameter and less) were observed to regress. To what extent regression occurred at an early stage was difficult to assess with certainty on naked eye examination alone.

*Histological appearances*

The earliest changes consisted of slight hyperplasia of the surface epithelium which was paralleled by similar hyperplasia of the epithelium of the upper part of the hair follicles (Fig. 1). This was often accompanied by dilatation of the upper part of the hair duct, the enclosed hair being surrounded by circumferential layers of keratin. Adjacent enlarged hair follicles soon coalesced (Fig. 2) the resultant cyst containing several hairs. One particularly large cyst of this type was seen to contain over 40 hairs (Fig. 3). The sequence of events leading to the development of the large cysts could often be traced from the keratin pattern within them. In each case keratin was initially laid down circumferentially round individual hairs. As fusion of the enlarged follicles occurred, groups of hairs came to be surrounded by larger rings of keratin produced by the epithelium of the cyst wall. Further coalescence resulted in the enclosure within circular deposits of keratin, of progressively larger aggregations of hairs. Fresh keratin was always laid down parallel to the cyst lining, and reflected the size of the cyst at the time of production of the particular sheet of keratin. Thus in the large cysts, the manner of development was often indicated by the character of the contained keratin. The cysts opened on to the surface through a pore of varying diameter. This fact no doubt explained both the apparent reduction in the number of hairs and the matting seen in the gross specimen, and also some of the small subepithelial nodules seen on naked eye examination. Where the cysts opened on to the surface through a wide mouth, large deposits of keratin were extruded (Fig. 4) and were seen in the gross as conical horns projecting from the surface. All the animals showed surface epithelial hyperplasia and changes in the hair follicles of at least the degree shown in Fig. 1.

In 40 of the rabbits the lesions were considerably more advanced. Changes leading to the development of macroscopic tumours tended to occur focally, and consisted of a further proliferation of either the surface epithelium, the epithelium lining the hair follicles or both in combination. Hyperplasia of the hair follicle epithelium sometimes occurred in the absence of much duct dilatation. Usually however, dilatation was marked and there was obvious cyst formation. Sebaceous glands were only occasionally visualised in relation to the cysts, and it seemed that the gland had either disappeared or had undergone squamous metaplasia and become incorporated into the cyst. Where follicular dilatation was less marked, the sebaceous glands were often more obvious and occasionally showed hyperplasia.

In the early stages epithelial hyperplasia in the hair follicles only involved the upper part. At a later stage it extended to involve the deepest parts of the follicle, the specialised features of which were no longer identifiable. In the latter case hairs were sometimes present in the upper part of a central core of keratin, which frequently showed the characteristic pattern already described. The keratin nearer the epithelial lining of such a cavity was laid down parallel to the surface and did not necessarily reflect the origin of the lesion in a number of hair follicles (Fig. 4, 5). This type of lesion presented the histological characteristics of molluscum sebaceum. Whether or not it showed smooth rolled edges in the gross appeared to depend on the degree of extension laterally under the normal skin. This was probably related to the size of both the antecedent pilosebaceous cyst and its surface opening. Those tumours which in the gross showed a large keratinous horn without raised edges in the surrounding epithelium showed essentially similar histological features, differing from the classical molluscum sebaceum only in showing a larger surface opening and relatively less lateral extension under the marginal epithelium (Fig. 4). The apparent ulceration seen in the gross was usually not accompanied by any breach in epithelial continuity. It was merely a reflection of loss of superficial keratin from these lesions.

Pure papillomas were much less frequent than lesions derived from the hair follicles. Tumours were classified as papillomas when they consisted of localised proliferation of the surface epithelium, with variable development of papillary folding. Hair follicle involvement was absent or inconspicuous (Fig. 6, 7). These tumours were always raised above the surface to some extent, and appeared either warty or plaque-like in the gross. Some of the warty lesions were seen on histological examination to present apparent papillary folding of the surface epithelium, which was in fact due to the presence of several adjacent pilosebaceous cysts (Fig. 10) and not to pure surface hyperplasia. The keratin pattern was useful in identifying this fact. Intermediate lesions showing both surface hyperplasia and involvement of the hair follicles were much more numerous than pure papillomas (Fig. 8, 9).

In all types of tumour very pronounced epithelial hyperplasia was seen in some of the larger lesions. The thickened epithelium was often thrown into papillary folds. At a later stage infiltrative growth in the vicinity of the tumour was evident. The cytological features of this type of growth were not distinguishable with certainty from those of the two proven carcinomas in the series. The fact that pseudoepitheliomatous hyperplasia occurred in relation to tumours which were known to be regressing was apt to influence the interpretation of similar appearances in connection with other tumours. While most of the tumours were clearly benign from the histological point of view, the exclusion of malignancy did not appear possible in some others. This difficulty was noted particularly in those lesions where the infiltrative growth was not sufficiently extensive to have brought about penetration of the cartilage or ulceration of the skin (undoubted indications of malignancy), especially when the tumour had not already commenced to regress at the time of examination. This histological interpretation explains low incidence of malignancy in these experiments as compared with the classical work of Berenblum (1945, 1949) using this carcinogen in rabbits.

This series was mainly concerned in ascertaining the development of lesions, and the exact sequence of events leading to regression was not studied in detail. Judging by the occurrence of naked eye diminution of some of the smaller nodules, it seemed that lesions could regress at an early stage. This fact was likewise suggested histologically by the presence of inflammation and epithelial degeneration in relation to some of the smaller pilosebaceous cysts, which were too small to be classified as tumours. Similar degeneration and inflammation were always associated with larger tumours which were known to be regressing at the time of examination.

### DISCUSSION

In the early stages of carcinogenic stimulation hyperplasia of the surface epithelium is closely paralleled by hyperplasia in the upper part of the pilosebaceous follicle. It therefore seems not unreasonable to suggest that the later development of the papilloma from surface epithelium is analogous to the growth of the molluscum sebaceum from the pilosebaceous follicle. Additional support is given to this suggestion by the occurrence of lesions obviously derived from both components. When the analogy between the two lesions is appreciated it becomes clear that to a large extent the " invasive growth " characteristic of molluscum sebaceum is merely a reflection of cellular proliferation in an unusual site. Pseudo-epitheliomatous hyperplasia may occur in both lesions, but it is likely to appear more sinister when situated deeply in the dermis in association with a molluscum sebaceum, than in relation to a more superficial papilloma.

The invaginated contours of the classical molluscum sebaceum appear to be produced by the coalescence of several pilosebaceous cysts, the orifice of which is frequently small by comparison with the diameter. In a few lesions in the present series cyst formation was less prominent in the hair follicles than hyperplasia, particularly in some of the compound lesions. The latter partook of the nature of both papilloma and molluscum sebaceum in showing proliferation of both surface and hair follicle epithelium (Fig. 8, 9). The contours of a lesion of this kind were dependent entirely on the relative preponderance of the two components and the tendency to cyst formation in the pilosebaceous follicles. At one end of the scale was a tumour showing mainly surface hyperplasia with relatively inconspicuous follicular hyperplasia and little tendency to cyst formation (Fig. 8). This type of lesion not infrequently showed hyperplastic sebaceous glands. At the other end of the scale was the classical molluscum sebaceum, the margins of which showed some superficial epithelial hyperplasia. In view of the fact that the lesions all appeared to arise in similar circumstances there seemed nothing to be gained from minute histological subdivision. Therefore tumours were classified as molluscum sebaceum or papilloma according to whether hyperplasia involved predominantly the hair follicles or the surface epithelium. It seems possible that these compound lesions in the rabbit may be similar in nature to some of the atypical papillomas seen in man. The latter often show the structure of a squamous papilloma but with the additional feature of epithelial hyperplasia in the hair follicles, sometimes accompanied by a minor degree of dilatation (Fig. 11).

In addition to the histological evidence suggesting that molluscum sebaceum develops from the hair follicles, circumstantial evidence was provided by the fact that it was never seen to occur in the centre of a disced area. The disc was initially filled with blood clot which became organised, the surface ultimately being covered by a simple epithelium devoid of skin appendages. Papillomas not infrequently occurred in this central area, but molluscum sebaceum was seen only at the margin where hair follicles remained. In this connection Ghadially (1958) mentions the fact that human molluscum sebaceum does not arise on the palm of the hand where hairs are absent. Experimentally the absence or relative diminution of hair follicles beneath the larger mollusca sebacea offered further confirmatory evidence of the participation of the hair follicles (Fig. 4, 5). An attempt is being made at present to produce molluscum sebaceum in the skin of rabbits following epilation by X-rays.

In view of the apparent participation of the pilosebaceous follicle in the development of experimental molluscum sebaceum, the retention of the latter term seems preferable to the other suggested alternatives. From the descriptive point of view the term kerato-acanthoma is non-specific and could equally well be applied to superficial papillomas.

The direct application of experimental findings in animals to clinical lesions in man is always hazardous. However, there is a considerable body of clinical and histological evidence to suggest that the lesion in man may represent a comparable process to that seen in the experimental animal. The human molluscum sebaceum occurs predominantly in those areas where skin cancers are most frequent. It also shows a predilection for a similar age group. It has been reported in tar, oil and arsenic workers and in people exposed to actinic radiation (Binkley and Johnson, 1955). The gross and histological appearances in man and animals closely parallel each other. An origin in the hair follicles has been postulated in man on histological evidence (Calnan and Haber, 1955). In the original English paper on molluscum sebaceum it was suggested that the lesion arose in a sebaceous cyst, a hypothesis which accords well with the findings in the present series. One wonders to what extent some of the simple epidermal cysts in man might be analogous to the experimental pilosebaceous cysts in representing a response to carcinogenic stimulation. The manner in which the keratin pattern indicated the contribution of individual hair follicles to the whole experimental lesion has already been described. This characteristic pattern was frequently, although not invariably present in the animal lesions. Fig. 12 is a human molluscum sebaceum showing a similar keratin pattern. Calnan and Haber's Fig. 6 likewise shows these features and Ghadially's Fig. 6 is also suggestive in this connection. The keratin pattern has not previously attracted attention in man, but in the light of experimental results it offers strong circumstantial evidence of the hair follicle origin of the human lesion. In some of the experimental lesions where large keratinous horns developed, the characteristic circular pattern persisted in the upper part of the horn, while the keratin nearest the epithelium was laid down parallel to it. It would thus seem that the earlier lesions are more likely to show the characteristic keratin pattern while the keratin is still contained within the cyst and has not yet been pushed too far distally.

The extent of epithelial hyperplasia in each molluscum sebaceum was varied. The impression was gained that papillary hyperplasia was most pronounced in those lesions showing a wide central pore. It seemed that in larger lesions opening through a small pore the pressure of retained keratin possibly limited papillary epithelial growth. The reverse picture was seen in those lesions showing a row of adjacent wide-marked pilosebaceous cysts (Ghadially's Type I kerato-acanthoma), the intervening walls between the cysts giving a markedly papillary appearance to the whole lesion (Fig. 10). In this type of tumour, the keratin pattern was found useful in identifying the nature of the lesion.

The hair growth cycle was at no stage taken into account in this experiment. However, two points in Whiteley's (1957) results appeared worthy of comment in relation to results obtained here. In the first place some of the lesions classified by him as papillomas would appear actually to consist of several adjacent wide mouthed pilosebaceous cysts. Ghadially (1958) has classified this lesion as a Type I kerato-acanthoma. Thus the stated differences in incidence of molluscum sebaceum and papilloma according to the stage in the hair cycle becomes less clearly defined. In the second place one wonders to what extent plucking of hairs may have acted as a Deelman phenomenon in the hair follicles. While the question of the relation of trauma to tumour growth is always a vexed one, the impression was gained in this series that the punching of discs in the ears did accelerate tumour growth. Accordingly it seems possible that repeated minor trauma involved in plucking hair could have acted in the same way.

### SUMMARY

Lesions resembling human molluscum sebaceum were produced in the skin of rabbits following painting with the carcinogen 9 : 10-dimethyl-1 : 2-benzanthracene. The genesis of these lesions from hair follicle epithelium is illustrated.

In addition squamous papillomas and compound tumours showing features of both papilloma and molluscum sebaceum were seen. It is suggested that the molluscum sebaceum is the hair follicle analogue of the superficial papilloma. Evidence is presented which suggests that molluscum sebaceum probably arises in man under similar circumstances to those obtaining experimentally. Lesions in man resembling the experimental compound tumours are also shown to occur.

I wish to acknowledge the technical assistance of Mr. A. C. Catchpole and Mr. E. Duff, Institute of Clinical Research, Middlesex Hospital, and Mr. L. Cantwell, Department of Surgery, University of Otago. Helpful advice and criticism was given by Dr. Peter Andrews, Bland-Sutton Institute of Pathology, Middlesex Hospital and Dr. Barbara Heslop, Department of Pathology, University of Otago. The photographs were prepared by Mr. F. H. Knight, University of Otago Medical School. Part of this work was carried out during the tenure of a Leverhulme Scholarship at the Institute of Clinical Research, Middlesex Hospital.

### REFERENCES

Beare, J. M.—(1953) *Brit. J. Surg.*, **41**, 167.

Berenblum, I.—(1945) *Cancer Res.*, **5**, 265.—(1949) *J. nat. Cancer Inst.*, **10**, 167.

Binkley, G. W. and Johnson, H. H.—(1955) *Arch. Derm. Syph., N.Y.*, **71**, 66.

Calnan, C. D. and Haber, H.—(1955) *J. Path. Bact.*, **69**, 61.

Fouracres, F. A. and Whittick, J. W.—(1953) *Brit. J. Cancer*, **7**, 58.

Ghadially, F. N.—(1958) *J. Path. Bact.*, **75**, 441.

Heslop, J. H.—(1956) Report to Institute of Clinical Research, Middlesex Hospital (unpublished).—(1958) *Proc. Univ. Otago med. Sch.*, **36**, 21.

Liban, E. and Lennox, B.—(1955) *Lancet*, i, 460.

MacCormac, H. and Scarff, R. W.—(1936) *Brit. J. Derm.*, **48**, 624.

Rook, A. J. and Whimster, I. W.—(1950) *Arch. belges. Derm.*, **6**, 137.

Rous, P. and Kidd, J. G.—(1939) *J. exp. Med.*, **69**, 399.

SEELIG, M. G. AND COOPER, Z. K.—(1933) *Amer. J. Cancer*, **17**, 589.
THOMSON, S.—(1958) *Ann. R. Coll. Surg. Engl.*, **22**, 382.
WHITELEY, H. J.—(1957) *Brit. J. Cancer*, **11**, 196.
WHITTLE, C. H. AND DAVIS, R. A.—(1957) *Lancet*, i, 1019.
WOGLOM, W. H.—(1926) *Arch. Path.*, **2**, 533.

---

### EXPLANATION OF PLATES

**FIG. 1.**—Hyperplasia of surface epithelium and epithelium lining the upper part of some hair follicles. × 15.

**FIG. 2.**—Further hyperplasia of hair follicle and surface epithelium with coalescence of adjacent follicles. × 10.

**FIG. 3.**—Large cyst produced by coalescence of numerous hair follicles. × 30.

**FIG. 4.**—Molluscum sebaceum with relatively wide surface opening and large keratinous horn. × 10.

**FIG. 5.**—Molluscum sebaceum with relatively narrow surface opening. × 6.

**FIG. 6.**—Papilloma presenting a plaque-like gross appearance. This area was disced and had healed with partial regeneration of cartilage. × 13.

**FIG. 7.**—Papilloma on healed disc, showing classical papillary pattern and wart-like gross appearance. × 11.

**FIG. 8.**—Compound tumour involving predominantly the surface epithelium. × 22.

**FIG. 9.**—Compound lesion with slightly more prominent hair follicle component. × 22.

**FIG. 10.**—Papillary appearance produced by several adjacent wide mouthed pilosebaceous cysts. × 27.

**FIG. 11.**—Warty lesion from man, present for some weeks. Hyperplasia of both surface and hair follicle epithelium, with circular keratin pattern on left. × 27.

**FIG. 12.**—Molluscum sebaceum in man showing circular arrangement of keratin reminiscent of experimental lesions. × 11.